# Cue Recognition and Integration – Eye Tracking Evidence of Processing Differences in Sentence Comprehension in Aphasia

**DOI:** 10.1371/journal.pone.0142853

**Published:** 2015-11-12

**Authors:** Rahel Schumacher, Dario Cazzoli, Noëmi Eggenberger, Basil Preisig, Tobias Nef, Thomas Nyffeler, Klemens Gutbrod, Jean-Marie Annoni, René M. Müri

**Affiliations:** 1 Perception and Eye Movement Laboratory, Departments of Neurology and Clinical Research, Inselspital, University Hospital Bern, and University of Bern, Bern, Switzerland; 2 Division of Cognitive and Restorative Neurology, Department of Neurology, Inselspital, Bern University Hospital, and University of Bern, Bern, Switzerland; 3 Gerontechnology and Rehabilitation Group, University of Bern, Bern, Switzerland; 4 University Hospital of Old Age Psychiatry and Psychotherapy, University of Bern, Bern, Switzerland; 5 ARTORG Center for Biomedical Engineering Research, University of Bern, Bern, Switzerland; 6 Neurology and Neurorehabilitation Center, Luzerner Kantonsspital, Luzern, Switzerland; 7 Neurology Unit, Laboratory for Cognitive and Neurological Sciences, Department of Medicine, Faculty of Science, University of Fribourg, Fribourg, Switzerland; 8 Center for Cognition, Learning and Memory, University of Bern, Bern, Switzerland; University of Leicester, UNITED KINGDOM

## Abstract

**Purpose:**

We aimed at further elucidating whether aphasic patients’ difficulties in understanding non-canonical sentence structures, such as Passive or Object-Verb-Subject sentences, can be attributed to impaired morphosyntactic cue recognition, and to problems in integrating competing interpretations.

**Methods:**

A sentence-picture matching task with canonical and non-canonical spoken sentences was performed using concurrent eye tracking. Accuracy, reaction time, and eye tracking data (fixations) of 50 healthy subjects and 12 aphasic patients were analysed.

**Results:**

Patients showed increased error rates and reaction times, as well as delayed fixation preferences for target pictures in non-canonical sentences. Patients’ fixation patterns differed from healthy controls and revealed deficits in recognizing and immediately integrating morphosyntactic cues.

**Conclusion:**

Our study corroborates the notion that difficulties in understanding syntactically complex sentences are attributable to a processing deficit encompassing delayed and therefore impaired recognition and integration of cues, as well as increased competition between interpretations.

## Introduction

Understanding spoken language is an ability we seem to use effortlessly on innumerable occasions during everyday life. It is thought that the language system uses semantic, syntactic, morphological, and prosodic cues in parallel in order to understand sentences, and that there is competition among potential alternative interpretations of a given sentence [1, 2].

A key aspect to correctly understand a sentence is the determination of who is doing what to whom. This process is known as thematic role assignment [3], where one part of the sentence receives the actor role and the other part is considered as the patient of an action or event. Cross-linguistic studies have shown that different cues are of varying importance for thematic role assignment in different languages. For instance, word order is most crucial in English, whereas case marking is most important in German [1].

Furthermore, predictive processes and heuristics (e.g. based on word order) are thought to play an important role in reducing the complexity of the task. The reliance on word order as a cue for thematic role assignment has also been described as actor-first strategy or heuristic [4], because the actor role is assigned to the first noun phrase encountered in a sentence (in languages with a canonical subject-verb-object word order). Sentences with non-canonical word order (i.e., the first noun phrase not being the actor) have been shown to increase processing demands in healthy subjects [4] and to produce miscomprehension in aphasic patients. Although traditionally associated with Broca’s aphasia, sentence comprehension difficulties have also been described in other aphasic syndromes [5, 6].

Patients’ difficulties in sentence comprehension have been attributed to specific impairments in different aspects of language processing, such as slowed syntactic [7] or lexical [8] activation, or impaired lexical integration [9]. Other accounts speak instead more generally of a “resource reduction” [10, 11] or a “limited resource availability” [12]. Another explanation holds that patients’ difficulties could stem from a deficit in recognizing cues and/or in integrating competing interpretations. Support for this view was found by Longoni [13], who examined a group of German speaking patients with varying aphasic syndromes with a sentence-picture matching task [14]. Among the different sentence types, there were canonical Subject-Verb-Object (SVO), non-canonical Object-Verb-Subject (OVS), and passive sentences. Patients were most impaired in processing OVS sentences, followed by passive ones, and only minimally impaired in processing SVO sentences. This replicated the findings from a previous study with a sentence-picture verification task [15]. The difficulties were attributed to a morphological problem (i.e., the case marking of the first noun’s article), and it was argued that morphological cues are more difficult to process because of their high confusability (low phonological and semantic salience, phonological similarity) that leads to a high competition between possible activations [13]. Therefore, patients tend to rely more on another cue, namely word order, which leads to a reversed thematic role interpretation of the sentence. In passive sentences, the word order vs. morphology cue conflict is easier to detect and resolve, because the cue is less local and more redundant (complex verb construction with preposition and case marking). These findings and interpretations are, however, based on behavioural or offline data that contain no information on the real-time or online processes leading to the observed result.

The measurement of eye movements (often referred to as eye tracking) is increasingly used to study sentence comprehension. Eye tracking is a valuable instrument to assess cognitive processes [16]. In fact, it provides a means to not only assess the end product of a process under study (i.e., accuracy, reaction time), but also to study the process itself by measuring the spatial and temporal distribution of fixations (i.e., the stable gaze position over some time) [17–19]. For instance, it could be shown that inspection and understanding of a visual scene are time-locked to the concurrent auditory input. This technique has been used in various experimental settings to study language comprehension in healthy populations [18–24] and, more rarely, in aphasic patients [9, 25–31].

The experimental paradigm used by Thompson and colleagues, entailing the presentation of pictured elements (e.g., a boy, a girl, a school, a door), is particularly suited to detect so called gap-filling or anticipatory fixations, i.e., fixations that fall on a target picture before the pictured element is mentioned in the sentence (see also [18, 32]). The simultaneous presentation of two or more pictured scenes (e.g., with reversed actor roles) has, in turn, the advantage that the stages of integration (i.e. the current interpretation of what has been heard so far) can be measured [10, 28]. For instance, Meyer et al. [28], found evidence for two different mechanisms in aphasic patients’ processing of passive sentences: a slow process leading to correct answers (successful integration), and a faster process resulting in incorrect responses (unsuccessful integration).

To the best of our knowledge, the only eye tracking studies in aphasic patients conducted in German so far are the ones by Hanne and colleagues [30, 31]. Similarly to Meyer et al. [28], two visual scenes with reversed thematic roles were shown simultaneously with auditory presented canonical (SVO) or non-canonical (OVS) sentences and evidence for delayed integration of morphological information in aphasic patients was found.

However, it is unclear to what extent the presentation of only two alternative interpretations, i.e., by showing the target picture and one distracter with reversed actor roles, influences sentence processing and task performance. It is, for instance, conceivable that the reversed actor role distracter would increase the probability of an actor-first interpretation in more complex, non-canonical sentences. We assume that the presentation of more distracter pictures would increase the number of possible interpretations of the verbal input. This, in turn, should lead to higher demands on competition resolution processes. Furthermore, based on the finding that sentences are processed incrementally (i.e., that each piece of information is instantaneously integrated upon hearing it [17]), it is possible to posit—for each moment in time—which picture versions can be dismissed as non-matching. We thus reason that the presentation of more than two picture stimuli allows drawing stronger conclusions concerning online processing (e.g., the application of actor-first strategies) and the source of potential errors. Additionally, we aimed at reducing the influence of possible difficulties in lexical activation [8], by repeatedly using a limited set of high frequency stimuli (e.g., man, woman, child). The stimuli used in previous studies [30, 31], for instance, contained less frequent nouns (e.g., smith, bricklayer, carpenter), which might increase processing load not only on the syntactical, but also on the lexical level.

Thus, in the present eye tracking study, we employ a sentence-picture matching task with canonical and non-canonical sentence types and the concurrent presentation of four pictured scenes (differing with respect to the mapping of the thematic roles and of an attribute). We aim at better understanding the mechanisms leading to impaired sentence comprehension, especially the proposed difficulties in the recognition and integration of cues [13]. Exploring these mechanisms is possible by means of the fine-grained temporal analysis of eye tracking.

Based on the extant literature, we can formulate several hypotheses concerning participants’ performance and their visual fixation patterns. Firstly, regarding patients’ performance, we expect that patients will solve non-canonical sentences less correctly than canonical sentences and that their performance for non-canonical sentences will be below healthy subject’s performance. Furthermore, we expect slower reaction times in patients compared to healthy subjects. Secondly, regarding the eye tracking data, we expect to find: 1) evidence for the application of actor-first strategies in both groups. This aspect would be reflected by an early fixation preference for the presumed actor; 2) evidence for patients’ difficulties in recognizing and integrating morphosyntactic cues, especially in OVS sentences where the cue is local. This difficulty would be reflected by diverging fixation patterns in the first parts of OVS sentences; 3) evidence for patients’ difficulties in integrating competing interpretations. This difficulty would be reflected by a delayed target preference (i.e., above chance level fixations on the target picture occurring later), especially in non-canonical sentences.

## Methods

### Participants

50 healthy subjects (mean age = 38.2 ± 15.1 (SD) years; 28 women) and 12 aphasic patients (mean age = 55 ± 15 years, 4 women; see Table 1 for further details) were included in the study. Diagnosis and classification of aphasia were based on neurological examination, standardized diagnostic procedures [33, 34] conducted by professional language therapists, and imaging data.

**Table 1:** Patients’ characteristics and accuracy in the sentence picture matching task.

			Stroke		Aphasia		Accuracy		
	Sex	Age	Months post	Type	Type	Severity	SVO	PASS	OVS
1	F	60	1.5	HS	Residual	minimal	1	0.92	0.75
2	F	52	73	HS	Residual	minimal	0.92	1	0.58
3	M	65	7	IS	Wernicke	moderate	1	0.08	0.25
4	F	42	3	HS	Broca	mild	0.83	0.67	0.58
5	M	18	3	IS	Anomic	moderate	1	0.83	0.58
6	M	59	31	IS	Anomic	mild	1	1	0.17
7	M	61	26	IS	Anomic	mild	1	0.75	0.42
8	F	44	5.5	IS	Broca	moderate	0.83	0.75	0.08
9	M	58	5	IS	Anomic	moderate	0.83	0.75	0.08
10	M	73	21	IS	Broca	mild	0.83	0.58	0.08
11	M	59	1	IS	Residual	minimal	1	1	0
12	M	69	5	IS	Broca	mild	0.92	0.75	0.75

F = Female, M = Male, HS = haemorrhagic stroke, IS = ischemic stroke, SVO = Subject-Verb-Object, PASS = passive, OVS = Object-Verb-Subject sentences

**Fig 1:**
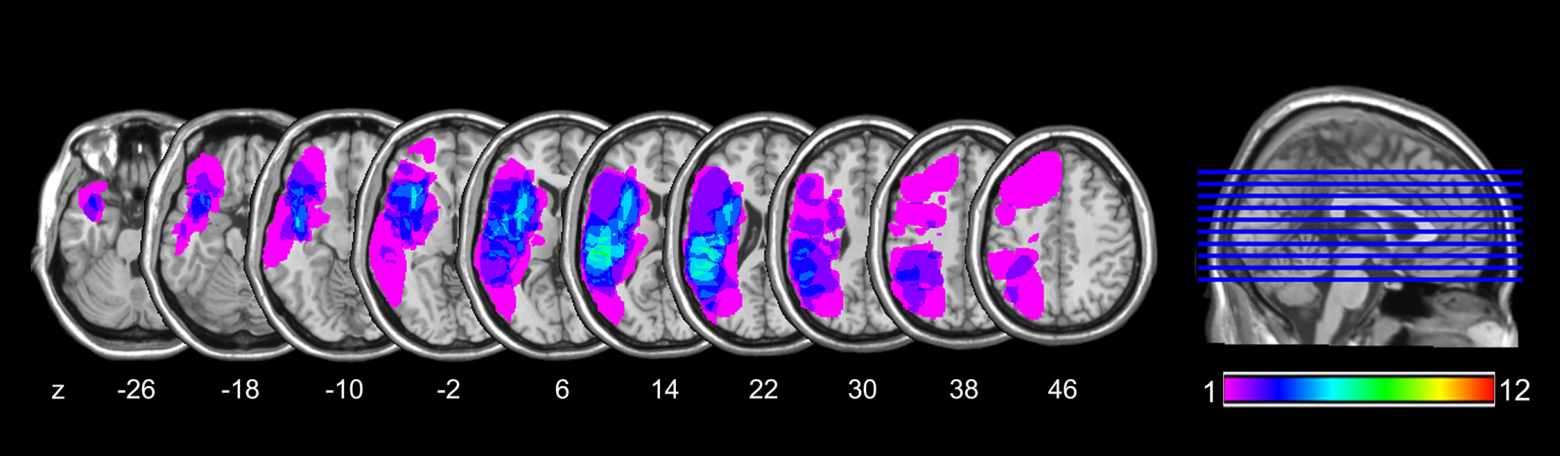
Lesion location and overlap maps for all patients. The z-position of each axial slice in Talairach stereotaxic space is indicated below the slices. Images are oriented according to non-radiological convention, i.e., left is left, and right is right.

Individual brain lesions (Fig 1) were traced from computer tomography or magnetic resonance imaging onto the standard Montreal Neurological Institute (MNI) brain using MRIcroN software [35].

All participants were native Swiss German speakers, and all had normal or corrected-to-normal vision and hearing. All participants gave written informed consent before the study. The investigation was carried out in accordance with the latest Declaration of Helsinki and was approved by the ethical committee of the State of Bern.

### Materials

The experiment consisted of a sentence-picture matching task. Forty-eight sentences were taken from a German sentence comprehension test (AAT-Supplement Satzverstehen; [14]). Semantically reversible sentences of three different types, i.e. with different case marking (nom = nominative, acc = accusative) and syntactical complexity, were used as target sentences:

1. Canonical, active sentences (Subject-Verb-Object, SVO) “Der Mann mit dem Korb fotografiert das Kind” The_nom_ man with the basket photographs the_nom/acc_ child «The man with the basket is photographing the child»
2. Non-canonical, passive sentences (PASS) “Das Kind mit dem Korb wird von dem Mann fotografiert” The_nom/acc_ child with the basket is by the man photographed «The child with the basket is being photographed by the man»
3. Non-canonical, active, topicalized sentences (Object-Verb-Subject, OVS) “Den Mann mit dem Korb fotografiert das Kind” The_acc_ man with the basket photographs the_nom/acc_ child «The child is photographing the man with the basket»

Relative sentences (e.g., “Das Kind, das den Mann fotografiert, trägt einen Korb”–The child that photographs the man has a basket) were used as filler trials. The SVO and OVS sentences were unambiguously case marked, and differed only regarding the case marking of the first word: determiner in nominative (in German “der”) for SVO, and in accusative (in German “den”) for OVS sentences. Due to the unambiguous accusative case marking in OVS sentences, the first noun phrase is incompatible with the actor role. In SVO and PASS sentences, however, the disambiguation between a subject-first vs. an object-first structure can only occur at the verb position. The sentences were recorded and spoken by a male native German speaker at a normal speaking rate (2.9 ± 0.4 words per second; [36]) and had a mean duration of 3 ± 0.4 seconds.

**Fig 2:**
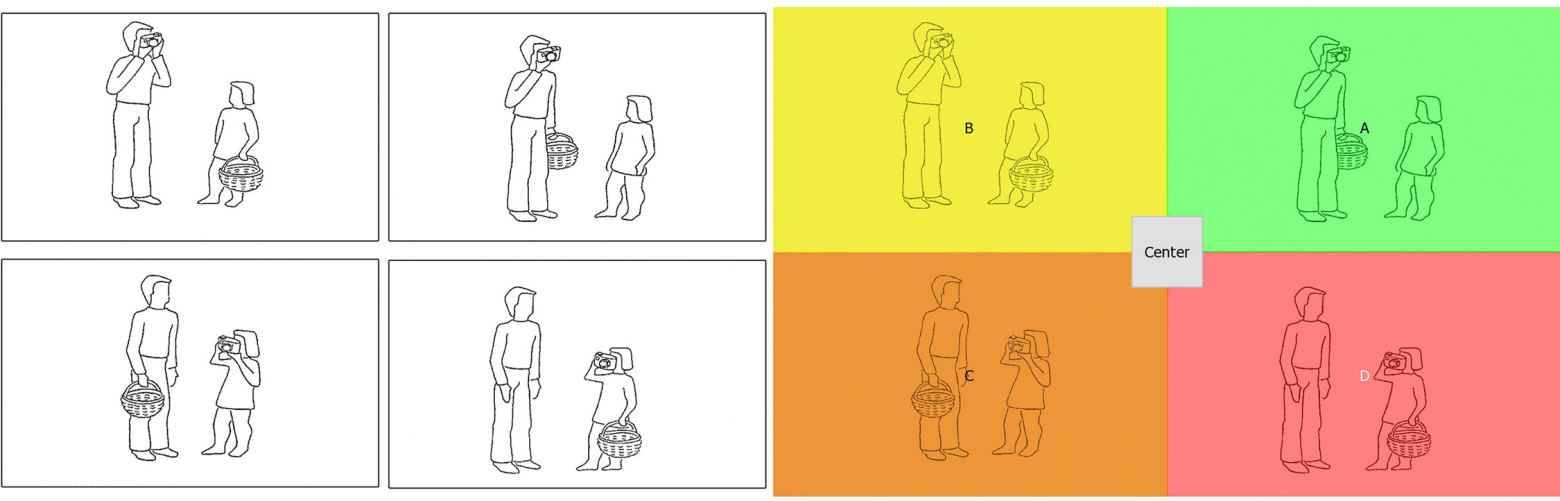
Picture set example and corresponding areas of interest used for the eye tracking analysis (colour-coded). For the sentence “the man with the basket photographs the child”, the top right version is the target picture (green), the top left picture is the first distractor (attribute wrong, yellow), the bottom left picture is the second distractor (actor wrong, orange), and the bottom right picture is the third distractor (attribute & actor wrong, red).

Pictures were taken from the same sentence comprehension test (AAT-Supplement Satzverstehen; [14]), and consisted of simple black-and-white line drawings. Every picture set consisted of four similar versions of a picture (e.g., a man/a child with a basket photographing a child/a man; see Fig 2 for an example). The four picture versions differed with respect to the mapping of the thematic roles and of the attribute. Accordingly, one of the four versions was the target picture (corresponding to the sentence content), and the other three versions served as distractors. In the first distractor, the attribute was with the wrong person (attribute wrong); in the second distractor, the actor was changed (actor wrong); and, in the third distractor, both the attribute and the actor did not correspond to the sentence (attribute & actor wrong).

### Procedure

The experiment was programmed using E-Prime 2.0 Pro software (Psychology Software Tools, Pittsburgh, PA). Instructions were given to the participants in written and oral form. Single spoken sentences were presented together with a set of four pictures (Fig 1). The participants were instructed to listen to the sentence, to explore the pictures, to choose the appropriate picture version, and to indicate their choice with a mouse click on the corresponding picture. Participants were instructed to respond as fast and as accurately as possible. In order to enforce a common starting point of visual exploration for all participants and trials, each trial began with a central fixation cross presented for 700ms, which participants were asked to fixate. The fixation cross was followed by the simultaneous presentation of the pictures and of the spoken sentence. At the same time, the mouse cursor always appeared at the centre of the screen. The spoken sentences were presented by headphones, and the pictures disappeared after the participant’s mouse click. Participants were allowed breaks between trials and could initiate the next trial with another mouse click.

The 12 picture sets were presented four times, each time with a different sentence, so that each picture version was the target picture once. The 48 trials were randomized, with the condition that consecutive trials did not involve the same picture set. Furthermore, the position of the target picture and of the distractors was counterbalanced across all trials and for each sentence type. Based on the latency and location of the mouse click, reaction time and accuracy were collected. Participants did not receive any feedback. The experiment lasted approximately 10 minutes for healthy subjects and up to 20 minutes for patients, the difference being mainly due to longer reaction times and longer breaks between trials in patients.

Eye movements were recorded using an integrated infrared eye-tracker (RED, SensoMotoric Instruments GmbH, Teltow, Germany) with a sampling rate of 60Hz, and a 22 inches screen with a resolution of 1680x1050 pixels. The distance to the screen was 70cm, thus resulting in a viewing angle of approximately 37.5 x 24°. The characters in the pictures subtended an approximate visual angle of 3–5° horizontally and 6–9° vertically. To calibrate the system, participants were asked to fixate a point that moved to five different locations on the screen. The calibration was followed by a validation procedure. If the validation procedure indicated a deviation greater than 1°, the calibration was repeated until this criterion was met.

### Data analysis

Accuracy of the responses was computed separately for each sentence type. Reaction time data were analysed for correct trials only. For accuracy and reaction time data, repeated-measures analyses of variance (ANOVA) were calculated using SPSS 21.0 and Statistica 6.0, with the three sentence types as within-subject factor, and group as between-subject factor. If the sphericity assumption was violated, a Greenhouse-Geisser correction was applied. For post-hoc tests, Fisher’s LSD-corrected t-tests were used.

To analyse fixation data, both spatial and temporal aspects were taken into account. The spatial distribution of fixations (defined as stable gaze position for at least 80ms) was analysed by using each of the four picture versions within a set as an area of interest (AOI, see Fig 2). This resulted in the following four AOIs per trial: A) target, B) attribute wrong, C) actor wrong, and D) attribute & actor wrong.

In order to consider the temporal aspect in the analysis, fixation data were segmented based on the different sentence parts in the auditory input. This was achieved by marking the beginning and the end of each particular sentence part (i.e., subject, attribute, verb, object) and the post-offset phase (until mouse click). When assigning these phases to the fixation data, a time lag of 200ms was added, in order to take into account the estimated time needed for auditory information processing and for the programming of a saccade [19]. Then, the number of fixations on each of the four AOIs was calculated for every sentence part, sentence type, and subject. This number was then divided by the total number of fixations on all AOIs in the respective sentence part. This resulted in the fixation proportion for each AOI, subject, and sentence type. Chance performance would result in a value of 0.25 for each AOI. For the sake of clarity, 0.25 was subtracted from every value. Hence, the resulting value indicates the deviation from chance level. A positive value indicates a fixation proportion above chance level, a value of 0 a fixation proportion at chance level and a negative value a fixation proportion below chance level. For each sentence type and sentence part, the mean proportions above chance level were submitted to a one-sample t-test, with a comparison value of 0. The level of statistical significance was set at 0.025 (one-tailed). As not many of the SVO and PASS trials were solved incorrectly, there was not enough data to analyse the fixation patterns for incorrectly solved SVO and PASS sentences. Furthermore, the same analyses were performed based on the cumulative fixation *duration*.

## Results

### Accuracy

**Fig 3:**
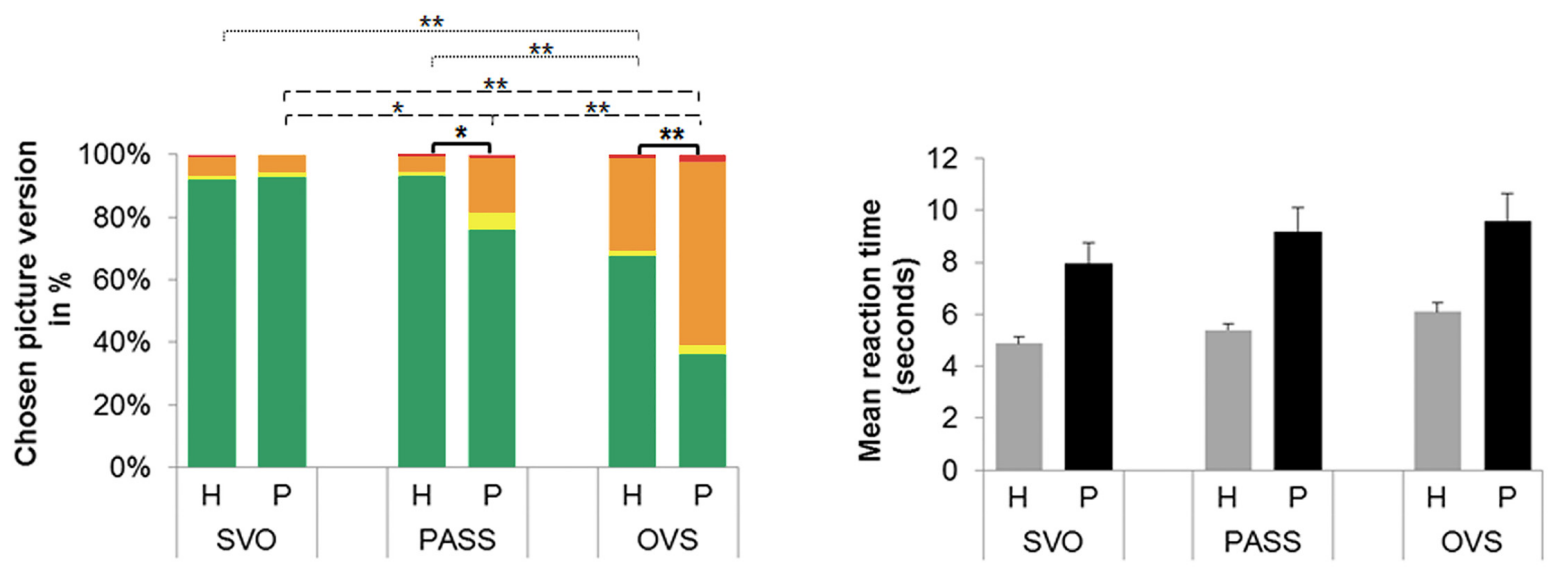
Accuracy rates and reaction times for the three sentence types in the two groups. On the left, accuracy rates (target chosen) and error types (one of the three distractors chosen) are depicted, together with significant post-hoc tests of the SentenceType*Group interaction. On the right, reaction times are depicted. SVO: Subject-Verb-Object, PASS: Passive, OVS: Object-Verb-Subject, H: Healthy, P: Patients, green bar: target (= accuracy), yellow bar: attribute wrong, orange bar: actor wrong, red bar: attribute & actor wrong, *: p < 0.05, **: p < 0.01, dotted lines: within healthy group, broken lines: within patient group, solid lines: between group comparisons.

Accuracy rates and error types for each sentence type are shown in Fig 3 (left). The repeated measures ANOVA revealed significant main effects of Sentence Type (F(1.3, 77.5) = 42.55, p < 0.01) and Group (F(1, 60) = 15.23, p < 0.01) as well as a significant interaction Sentence Type*Group (F(1.3, 77.5) = 6.22, p < 0.01). Post-hoc tests showed that, both in the group of healthy controls and the group of aphasic patients, OVS sentences were solved less correctly (Healthy: 68 ± 4.8%; Patients: 36 ± 8.0% correct) than PASS (93 ± 1.1%, p < 0.01; 75.5 ± 7.3%, p < 0.01) and SVO (92 ± 1.2%, p < 0.01; 93 ± 2.2%, p < 0.01) sentences. Furthermore, patients committed more errors on PASS sentences than on SVO sentences (p < 0.05), which was not the case for healthy controls (p = 0.78). Between-group comparisons for each sentence type revealed significant differences for OVS sentences (p < 0.01), and for PASS sentences (p < 0.05), but not for SVO sentences (p = 0.97).

### Reaction times

Mean reaction times for each sentence type are shown in Fig 3 (right). The repeated measures ANOVA revealed significant main effects of Sentence Type (F(2, 114) = 10.69, p < 0.01) and Group (F(1, 57) = 28.68, p < 0.01), but no significant interaction Sentence Type*Group (F(2, 114) = 1.14, p = 0.32). Irrespective of group, SVO sentences elicited shorter reaction times (5498 ± 296 ms) than PASS sentences (6175 ± 344 ms, p = 0.01) and than OVS sentences (6743 ± 376 ms, p < 0.01), but there was no significant difference between PASS and OVS sentences (p = 0.181). Patients generally responded more slowly than healthy subjects (Healthy: 5427 ± 238 ms, p < 0.01; Patients: 8845 ± 821 ms).

### Eye tracking data

In the following, we first describe the healthy subjects’ fixation patterns and then the patients’ ones, separately for every sentence type. All p-values are < 0.01, unless stated otherwise.

#### SVO sentences

**Fig 4:**
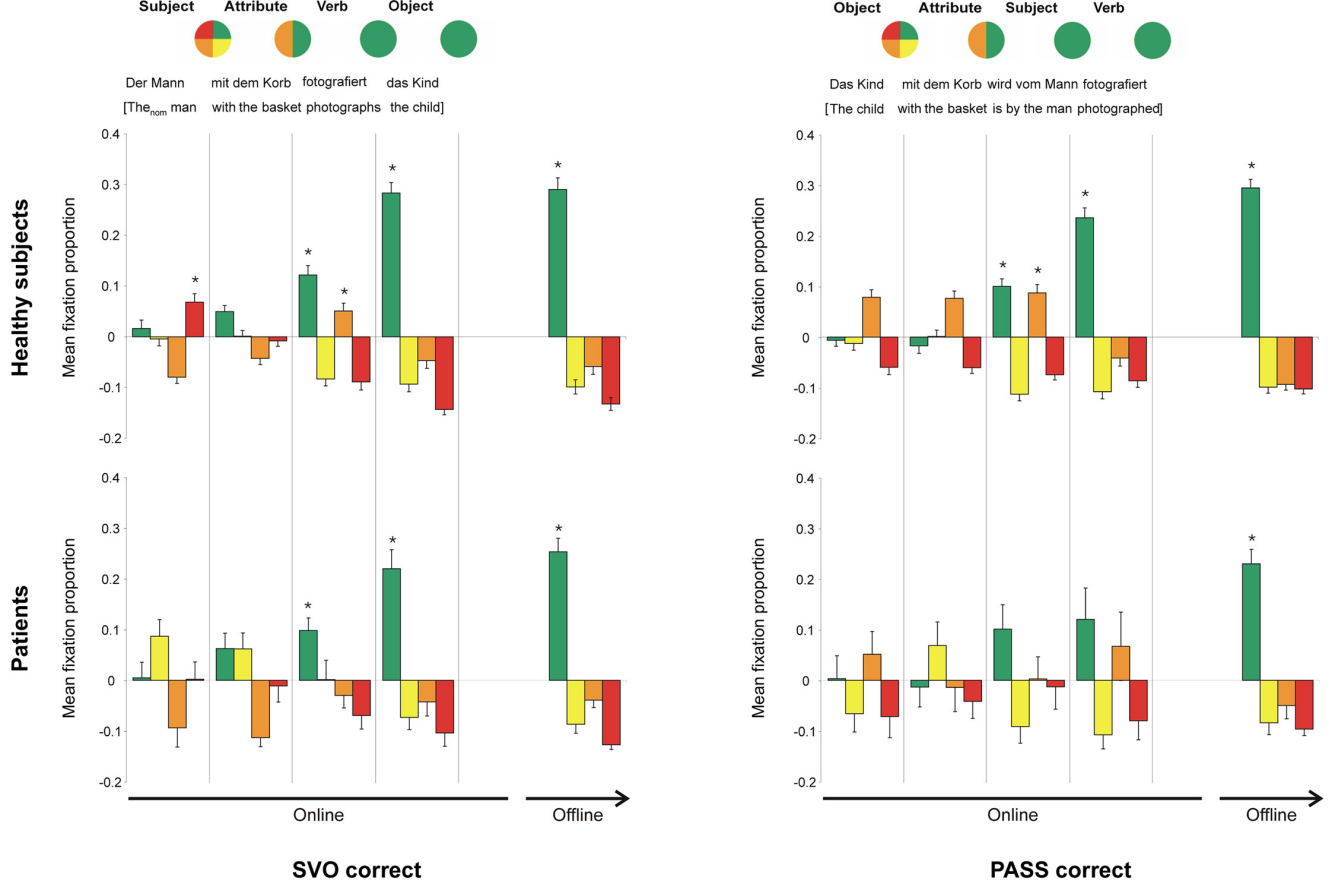
Fixation proportions on the four areas of interest for correctly solved SVO and PASS sentences. The circles in the top row indicate the matching picture versions, depending on the sentence’s content heard so far, at different time points. A quartered circle indicates that all versions would be suitable. A circle cut in half indicates that only two picture versions remain as possible targets. The colour indicates which distractor would be appropriate besides the target. A completely green circle indicates that only the target matches the sentence’s content. An example sentence, together with a translation, is given below the circles. In the graphs, the mean fixation proportions above/below chance level for each sentence part (online), as well as for the offline phase (until mouse click), is depicted. Green: target, yellow: attribute wrong, orange: actor wrong, red: attribute & actor wrong: error bars: standard error of the mean; *: p < 0.025 (fixation proportion above chance level).

Healthy subjects (Fig 4, top left) showed a significant preference in fixating the target and, to a lesser extent, also the “actor wrong” picture during the verb sentence part. The “actor wrong” picture fixations ceased during the object sentence part, and the target preference increased over time, becoming most prominent in the offline phase (i.e., after the end of the spoken sentence until the response). Patients (Fig 4, bottom left) showed a similar pattern but did not preferentially fixate the “actor wrong” picture during the whole trial.

#### PASS sentences

Healthy subjects (Fig 4, top right) showed a pattern similar to the one of the SVO sentences when PASS sentences were processed. They showed a preference in fixating the target and the “actor wrong” picture, which was refined towards the target picture upon hearing the subject sentence part (that, in this sentence type, contains the auxiliary verb structure indicating that it is a passive sentence). Patients (Fig 4, bottom right) showed a slightly different pattern. Comparable to the healthy subjects, they showed a preference in fixating the target picture from the subject sentence part onwards. This preference was, however, not significantly above chance level until after sentence offset. In addition to the target picture, the “actor wrong” picture was also fixated more often, but this increase appeared later than in healthy subjects, and it was again not significant.

#### OVS sentences

**Fig 5:**
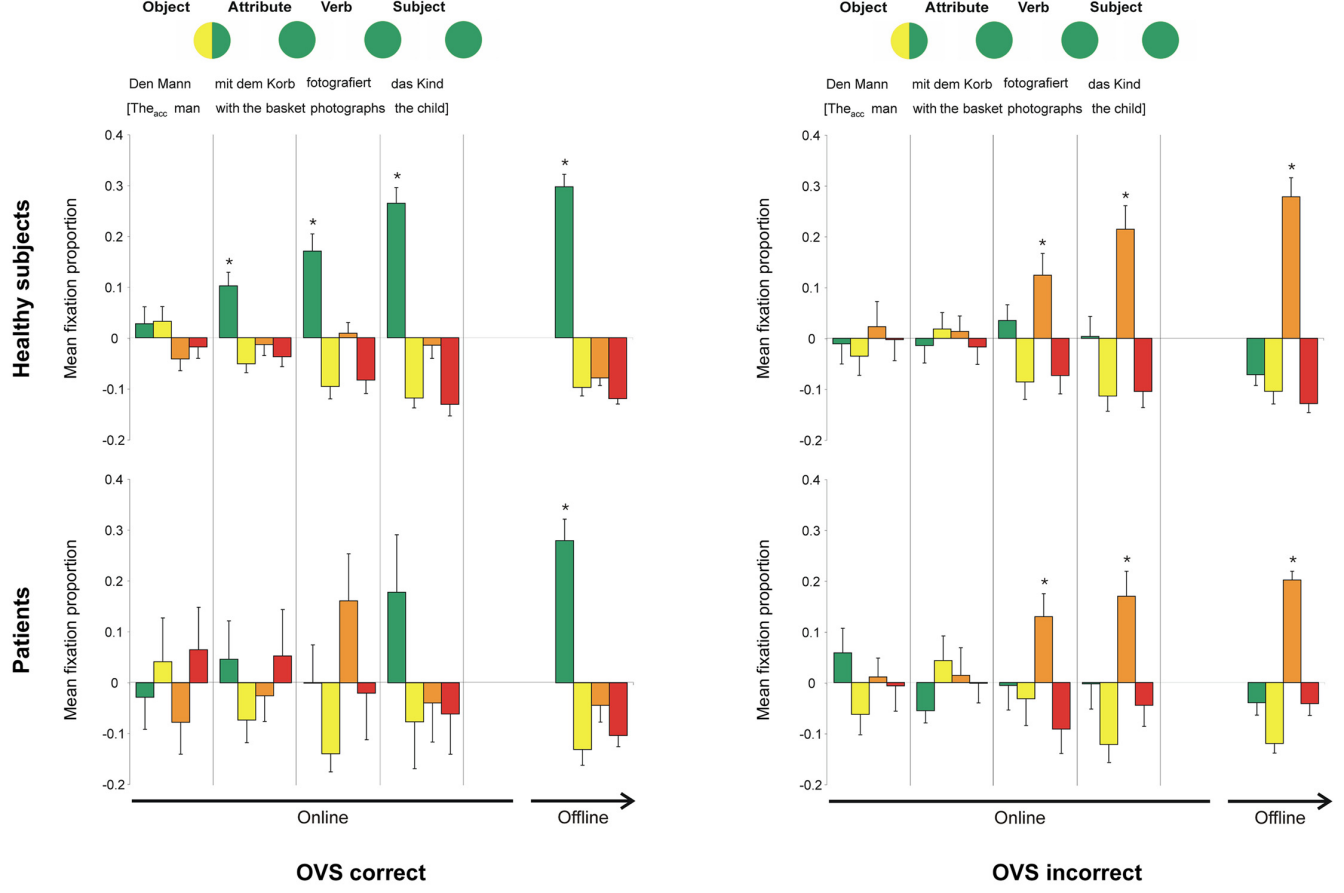
Fixation proportions on the four areas of interest for correctly and incorrectly solved OVS sentences. The circles in the top row indicate the matching picture versions, depending on the sentence’s content heard so far, at different time points. As the unambiguous accusative case marking allowed immediate exclusion of the child as an actor, only two picture versions (indicated by a circle cut in half, the colour corresponding to the distractor that would be appropriate besides the target) remain as possible targets after the first sentence part. Upon hearing the attribute, the target could already be identified (indicated by a fully green circle). An example sentence, together with a translation, is given below the circles. In the graphs, the mean fixation proportions above/below chance level for each sentence part (online) as well as for the offline phase (until mouse click) is depicted. Green: target, yellow: attribute wrong, orange: actor wrong, red: attribute & actor wrong; error bars: standard error of the mean; *: p < 0.025 (fixation proportion above chance level).

In correctly solved sentences, the fixation pattern of healthy subjects (Fig 5, top left) reflected the fact that the unambiguous case marking allowed correct identification of the target picture already upon hearing the attribute. Thus, a significant preference in fixating the target picture evolved already during the attribute sentence part, and increased until subjects indicated their choice with the mouse click. Patients (Fig 5, bottom left) did not fixate any picture version significantly above chance level while listening to the sentence, but showed a significant preference for the target picture during the post-offset phase. Additionally, in contrast to the other sentence types, their qualitative pattern differed from that of healthy subjects. Patients appeared to fixate the “actor wrong” picture during the verb sentence part, and switched to the target picture only during the subject sentence part. Although the increased fixations of the “actor wrong” distracter did not reach statistical significance, this pattern will be discussed in more detail in the discussion section, as it is relevant for understanding the mechanisms underlying impaired comprehension.

In incorrectly solved OVS sentences (Fig 5, right), healthy controls and patients showed a very similar pattern, with a preference in fixating “actor wrong” picture, starting during the verb sentence part and increasing until the mouse click. This pattern strongly resembled the one found for SVO sentences, with fixation proportions significantly above chance level (patients p = 0.029) from the verb sentence part onwards and increasing until the mouse click.

The analyses based on the fixation *duration* data yielded very similar results. These are depicted in S1 Fig.

## Discussion

By using a sentence-picture matching task, we aimed at elucidating the mechanisms of successful and unsuccessful processing of differentially complex German sentences in healthy subjects and patients with aphasia. A design with four very similar pictured scenes to choose from allowed to increase competition among conflicting interpretations, and thus to perform a more detailed analysis of errors. This, in turn, facilitated the detection of difficulties in cue recognition and integration, and of the application of actor-first strategies.

As expected, aphasic patients showed generally slower reaction times and performed worst in the non-canonical sentence types. Over all sentence types and in both groups, the distractor picture with the reversed actor roles was chosen most often when an error was committed.

Healthy subjects and patients performed at ceiling for SVO sentences. Importantly, the high accuracy rates in this simpler sentence type indicate that the presentation of four pictured scenes to choose from did not make the task too complex for patients. Thus, the task provides a reliable way to measure online processing. Regarding fixation patterns in healthy subjects, a preference for the target picture evolved over time, and became significant already before sentence offset. This fixation pattern paralleled more or less the theoretical sequence of distractor elimination. Patients showed a target fixation pattern that was qualitatively comparable to the one of healthy subjects. However, in contrast to the healthy subjects, they did not show a fixation proportion significantly above chance level on the distracter with reversed actor roles. This could, in line with Hanne and colleagues [31] be interpreted as a sign for a stronger prediction of an SVO structure.

In PASS sentences, comparably to SVO sentences, healthy subjects showed an early narrowing down to two competitors. Furthermore, there was evidence of an actor-first strategy with increased fixation proportions on the picture with the reversed actor role during the first three sentence parts. This preference decayed over time, and was replaced by a significant target preference as soon as the auxiliary verb and the second noun phrase were presented.

Aphasic patients showed no obvious sign of an actor-first strategy in passive sentences. Based on their fixation patterns in OVS sentences, we would have expected this strategy to appear in the subject sentence part. This is exactly where several cues (auxiliary verb, preposition) indicate the passive structure. As these cues are more global and redundant, their processing is facilitated [13] and they could thus be recognized and integrated more rapidly. This would collide with the emergence of the initial actor-first strategy. Another interpretation, as proposed by Hanne and colleagues [31], is that patients would adopt a “wait and see”-strategy, due to the ambiguous case-marking at the beginning of the sentence. Moreover, patients tended to have an increase in fixations on the picture with the reversed actor role during the verb sentence part, the last part in this sentence type. This could be interpreted as a sign of late competition between the two interpretations [26]. The finding of a delayed target preference (appearing only after sentence offset) in patients is in line with the findings of Meyer and colleagues [28], and also compatible with an integration deficit account or a slowed processing account. However, this observed delay has to be interpreted with caution, because the two groups differed regarding sample size and thus statistical power.

So far, eye tracking studies reported comparisons *between* correctly and incorrectly solved trials, either within or across groups of patients and healthy subjects [9, 25, 26, 28, 30]. In this context, the assessment of potential differences between groups *within* correctly solved trials, as well as *within* incorrectly solved trials, is of great relevance, since it can reveal differences in the mechanisms underlying sentence (mis)comprehension. This latter comparison has—to the best of our knowledge—not been assessed yet. The fact that the healthy subjects in our study committed a relatively high number of errors in OVS sentences enabled us to assess this novel aspect. We will thus firstly discuss the two groups’ fixation patterns for incorrectly solved trials.

The fixation patterns in the two groups did not differ for incorrect sentences. In incorrectly solved trials, both groups seem to have processed OVS sentences as if these were SVO sentences, since the fixation patterns in the two cases were strikingly similar. Thus, when choosing the wrong answer, both, healthy subjects and aphasic patients, misinterpreted the sentences in a similar way, but this mistake occurred more frequently in patients.

Regarding the correctly solved OVS sentences, a different pattern was observed. Healthy subjects showed a strong preference for the target picture already after hearing the first part of the sentence. Thus, they seemed to immediately recognize and integrate the morphosyntactic cue (indicating that the first noun phrase was not the actor of the sentence) into the ongoing processing. However, this did not lead to speeded responses, as the reaction times for OVS sentences were significantly higher than the ones for SVO sentences. This observation was also reported in earlier studies, and was attributed to increased processing costs [31].

Patients, in turn, developed a preference for the target picture only at the end of the sentence. They seemed, at least in part, to begin with an SVO interpretation (hence the preference for the “actor wrong” picture during the verb sentence part, although this trend did not reach statistical significance), which could be interpreted as a delayed actor-first strategy. This was followed by a revision process, which started in the last part of the sentence and led to the correct answer. Thus, in contrast to healthy subjects, patients firstly showed no immediate recognition and integration of the morphosyntactic cue, but instead there is evidence that an actor-first strategy might have been applied; and, secondly, they showed evidence for a revision process. The lack of immediate morphosyntactic cue integration is in line with Longoni’s [13] proposition of a morphosyntactic retrieval deficit in patients, and also with the result of the recent study by Hanne and colleagues [31].

Taken together, no differences regarding target fixation patterns were found between groups in SVO sentences. In PASS sentences, patients’ fixation patterns were not indicative of an actor-first strategy, but of increased competition between interpretations towards the end of the sentence. Finally, in OVS sentences, healthy subjects either: 1) did not recognize the morphological cue at the beginning of the OVS sentence, and interpreted it as if it was an SVO sentence; or, 2) they recognized and immediately integrated the morphological cue, which led to a correct interpretation. In contrast to healthy subjects, patients never exhibited immediate recognition and integration of the morphological cue in OVS sentences, but still reached a correct interpretation of the sentence in some trials. We attribute the differences between healthy subjects and aphasic patients in the processes leading to the correct interpretation of OVS sentences to delayed cue recognition and/or an integration deficit due to increased competition with an SVO interpretation in patients. In line with Meyer et al. [28], and in contrast to Hanne and colleagues [31], unsuccessful integration processes seem to be faster than successful ones. In other words, aphasic patients seem to differ from healthy controls regarding the way they “get it right” and, at least for OVS sentences, not regarding the way they “get it wrong”. To our knowledge, the latter has not been shown before.

Future studies should focus on investigating the relationship between executive functions and comprehension processes, since prediction and conflict resolution processes seem to play a crucial role in sentence comprehension. Moreover, comprehension deficits for non-canonical sentences have been described in other populations, such as children [37], patients with dementia of the Alzheimer type [38–40], and patients with Parkinson’s disease [41, 42], and these deficits are commonly attributed to decreased executive functions (working memory, set-shifting, inhibition, or cognitive control capabilities) [43]. The influence of these functions on aphasic comprehension impairments has not been intensively studied yet, except for working memory [44]. For instance, performance in the Stroop task (taken as a measure of cognitive control) was indeed correlated with performance in a sentence comprehension task in healthy adults [45], and the relevance of domain-general cognitive control in post-stroke aphasia was also demonstrated in a recent functional magnetic resonance imaging study [46]. Deficits in cognitive control could affect the overreliance on specific cues (e.g., word order in this study), as well as conflict detection and resolution processes.

## Conclusion

In our analysis of sentence processing, eye tracking data yielded evidence for patients’ deficits in recognizing and integrating morphological cues. Our study corroborates the notion that difficulties in understanding syntactically complex sentences are attributable to a processing deficit encompassing delayed and therefore impaired recognition and integration of cues, as well as increased competition between reversed actor role interpretations.

## Supporting Information

S1 Fig Proportions of fixation duration on the four areas of interest for the three sentence types. (TIF)
