# Supplementary figures and images for: Cue Recognition and Integration – Eye Tracking Evidence of Processing Differences in Sentence Comprehension in Aphasia
Source: PLoS One. 2015 Nov 12;10(11):e0142853. doi: 10.1371/journal.pone.0142853 (PMC4642964; doi:10.1371/journal.pone.0142853)

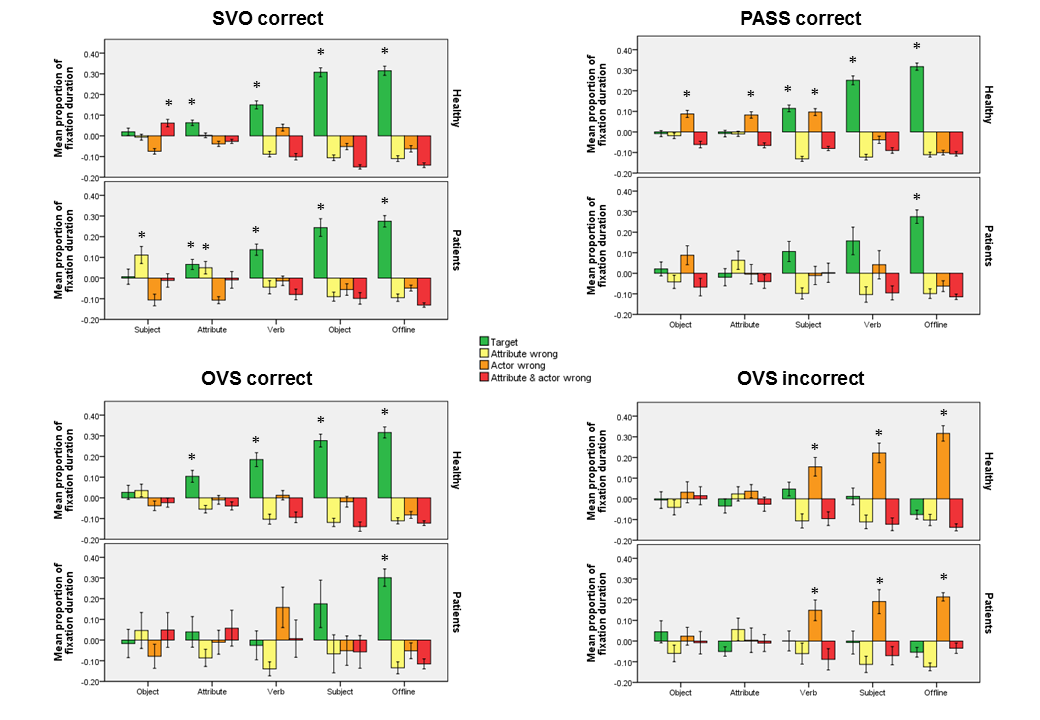

Supplement: S1 Fig — (TIF) [file pone.0142853.s001.tif]
